# Supplementary material for: Ultra-Processed Food Intake as an Effect Modifier in the Association Between Depression and Diabetes in Brazil: A Cross-Sectional Study
Source: Nutrients. 2025 Jul 28;17(15):2454. doi: 10.3390/nu17152454 (PMC12348790; doi:10.3390/nu17152454)
Supplement: Supplementary file 1 [file nutrients-17-02454-s001.zip › nutrients-3699443-supplementary.pdf]

Dietary Pattern Categories	Food Groups	Low intake threshold	Low Consumption	Medium Consumption	High Consumption	Mean VIF
**Plant-based whole food**	beans<br>vegetables<br>natural fruit juice<br>fruits	< 5x<br>< 7x<br>< 2x<br>< 7x	All food groups were recognized as low intake	Has at least one food group recognized as high intake but not all	All food groups were recognized as high intake	1.15
**Animal-based whole food**	red meat<br>chicken<br>fish<br>milk	< 3x<br>< 3x<br>< 2x<br>< 7x	All food groups were recognized as low intake	Has at least one food group recognized as high intake but not all	All food groups were recognized as high intake	1.00
**UPF - classification method 1 (UPF1)**	artificial fruit juice<br>soda<br>sweets<br>substitutive meals	0<br>0<br>< 2x<br>0	All food groups were recognized as low intake	N/A	Has at least one food groups recognized as high intake	1.04
**UPF - classification method 2 (UPF2)**	artificial fruit juice<br>soda<br>sweets<br>substitutive meals	0<br>0<br>< 2x<br>0	Has at least one food groups recognized as low intake	N/A	All food groups were recognized as high intake	1.04

Supplementary Table S1. Dietary patterns classification according to weekly consumption of food groups.
Abbreviation: UPF: ultra-processed food; N/A: not applicable; VIF: Variance inflation factor.

Odds ratio (95% CI)	UPF1					UPF2				
	Unadjusted	Basic	Interactive	Stratified - low consumption	Stratified - high consumption	Unadjusted	Basic	Interactive	Stratified - low consumption	Stratified - high consumption
N	**81,524**	**62,187**	**62,187**	**19,562**	**42,625**	**81,524**	**62,187**	**62,187**	**59,732**	**2,455**
R^2 (%, Adjusted)	3.45	15.71	15.74	11.52	14.94	0.58	14.77	14.83	14.41	20.88
**1.Depression (no as reference)**										
Yes	1.515 (1.408, 1.629)***	1.258 (1.064, 1.489)**	1.035 (0.836, 1.281)	1.045 (0.844, 1.294)	1.401 (1.107, 1.774)**	1.529 (1.422, 1.643)***	1.251 (1.059, 1.478)**	1.209 (1.021, 1.432)*	1.211 (1.023, 1.434)*	3.551 (1.394, 9.046)**
**2.Diatery patterns**										
UPF consumption (low as reference)										
high	0.370 (0.352, 0.389)***	0.547 (0.495, 0.605)***	0.522 (0.469, 0.582)***	N/A	N/A	0.371 (0.309, 0.446)***	0.796 (0.571, 1.109)	0.684 (0.474, 0.987)*	N/A	N/A
Plant-based food consumption (low as reference)										
intermediate	N/A	1.030 (0.825, 1.286)	1.033 (0.827, 1.289)	1.102 (0.831, 1.461)	1.007 (0.749, 1.353)	N/A	1.058 (0.851, 1.315)	1.055 (0.848, 1.312)	1.076 (0.865, 1.339)	0.860 (0.315, 2.348)
high	N/A	0.932 (0.722, 1.203)	0.934 (0.724, 1.205)	1.062 (0.767, 1.470)	0.845 (0.591, 1.207)	N/A	1.011 (0.786, 1.299)	1.007 (0.783, 1.295)	1.049 (0.815, 1.351)	0.062 (0.012, 0.324)**
Animal-based food consumption (low as reference)										
intermediate	N/A	1.062 (0.827, 1.364)	1.058 (0.825, 1.358)	1.178 (0.837, 1.658)	0.986 (0.695, 1.400)	N/A	1.019 (0.795, 1.306)	1.015 (0.792, 1.301)	1.015 (0.788, 1.306)	1.061 (0.286, 3.933)

high	N/A	1.159 (0.816, 1.644)	1.153 (0.813, 1.636)	1.227 (0.732, 2.054)	1.125 (0.701, 1.805)	N/A	1.101 (0.777, 1.562)	1.101 (0.777, 1.562)	1.112 (0.780, 1.586)	0.699 (0.095, 5.151)
**3. Health**										
Smoking status (never as reference)										
ever	N/A	1.215 (1.093, 1.352)***	1.215 (1.093, 1.351)***	1.304 (1.129, 1.506)***	1.151 (0.989, 1.339)	N/A	1.206 (1.085, 1.340)**	1.208 (1.087, 1.343)***	1.207 (1.085, 1.342)**	1.327 (0.625, 2.816)
Alcohol consuming last month (no as reference)										
Yes	N/A	0.759 (0.669, 0.860)***	0.758 (0.669, 0.859)***	0.638 (0.538, 0.757)***	0.845 (0.711, 1.004)	N/A	0.744 (0.658, 0.843)***	0.743 (0.656, 0.841)***	0.749 (0.661, 0.848)***	0.584 (0.258, 1.322)
Obese (no as reference)										
Yes	N/A	2.050 (1.835, 2.289)***	2.050 (1.835, 2.289)***	1.971 (1.696, 2.291)***	2.091 (1.790, 2.443)***	N/A	2.051 (1.838, 2.289)***	2.051 (1.837, 2.289)***	2.048 (1.833, 2.289)***	2.086 (1.026, 4.244)*
Sufficient physical activity (Yes as reference)										
No	N/A	1.134 (1.009, 1.275)*	1.137 (1.011, 1.279)*	1.201 (1.030, 1.400)*	1.084 (0.915, 1.286)	N/A	1.078 (0.961, 1.211)	1.080 (0.962, 1.212)	1.081 (0.963, 1.215)	0.919 (0.414, 2.040)
**4.Demographic and socioeconomic status**										
Sex (male as reference)										

Female	N/A	0.908 (0.813, 1.013)	0.905 (0.811, 1.010)	0.808 (0.702, 0.930)**	0.974 (0.830, 1.142)	N/A	0.931 (0.835, 1.038)	0.931 (0.835, 1.038)	0.927 (0.830, 1.034)	1.118 (0.548, 2.280)
Region (north as reference)										
Northeast	N/A	1.136 (0.977, 1.320)	1.137 (0.978, 1.321)	1.172 (0.962, 1.427)	1.088 (0.865, 1.370)	N/A	1.136 (0.979, 1.318)	1.136 (0.979, 1.318)	1.135 (0.977, 1.318)	1.017 (0.257, 4.024)
Southeast	N/A	1.386 (1.183, 1.623)***	1.388 (1.185, 1.627)***	1.403 (1.132, 1.738)**	1.377 (1.091, 1.739)**	N/A	1.249 (1.068, 1.461)**	1.252 (1.071, 1.465)**	1.259 (1.075, 1.474)**	0.854 (0.262, 2.785)
South	N/A	1.212 (1.018, 1.442)*	1.214 (1.020, 1.444)*	1.092 (0.854, 1.395)	1.282 (1.002, 1.640)*	N/A	1.093 (0.921, 1.297)	1.094 (0.922, 1.298)	1.093 (0.921, 1.297)	0.865 (0.236, 3.168)
Central	N/A	1.280 (1.066, 1.536)**	1.283 (1.069, 1.539)**	1.622 (1.254, 2.097)***	1.067 (0.819, 1.389)	N/A	1.189 (0.993, 1.423)	1.188 (0.992, 1.422)	1.203 (1.004, 1.443)*	0.475 (0.110, 2.055)
Highest education obtained (Elementary school- as reference)										
High school	N/A	0.723 (0.636, 0.823)***	0.723 (0.636, 0.823)***	0.707 (0.596, 0.839)***	0.737 (0.613, 0.885)**	N/A	0.724 (0.637, 0.822)***	0.724 (0.637, 0.822)***	0.705 (0.619, 0.802)***	1.864 (0.843, 4.121)
University+	N/A	0.560 (0.480, 0.655)***	0.560 (0.480, 0.654)***	0.571 (0.457, 0.712)***	0.554 (0.446, 0.687)***	N/A	0.568 (0.487, 0.664)***	0.568 (0.487, 0.664)***	0.550 (0.470, 0.643)***	1.682 (0.662, 4.273)
Residence (urban as reference)										
Rural	N/A	0.705 (0.622, 0.800)***	0.703 (0.620, 0.798)***	0.697 (0.589, 0.825)***	0.704 (0.583, 0.849)***	N/A	0.725 (0.639, 0.821)***	0.723 (0.638, 0.819)***	0.719 (0.634, 0.816)***	0.961 (0.362, 2.551)

Marital status (married as reference)											
	Other	N/A	0.877 (0.789, 0.976)*	0.879 (0.790, 0.977)*	0.957 (0.835, 1.098)	0.835 (0.715, 0.974)*	N/A	0.861 (0.775, 0.957)**	0.863 (0.776, 0.958)**	0.871 (0.783, 0.969)*	0.665 (0.353, 1.253)
Age		N/A	1.051 (1.048, 1.055)***	1.051 (1.048, 1.055)***	1.044 (1.039, 1.050)***	1.055 (1.051, 1.060)***	N/A	1.054 (1.051, 1.058)***	1.054 (1.051, 1.058)***	1.054 (1.050, 1.057)***	1.078 (1.054, 1.102)***
**5. Interactive term**		N/A	N/A	1.379 (1.009, 1.885)*	N/A	N/A	N/A	N/A	2.900 (1.197, 7.028)*	N/A	N/A

Supplementary Table S2. Results of preliminary logistic regression.

Abbreviation: CI: confidence interval; UPF1: ultra-processed food consumption classification method 1; UPF2: ultra-processed food consumption classification method 2; N/A: not applicable.

Notes

* ***P*** <0.05; ** ***P*** <0.01; *****P*** <0.001.

Model	UPF1				UPF2			
	**low consumption, 18-59 years**	**low consumption, 60+ years**	**high consumption, 18-59 years**	**high consumption, 60+ years**	**low consumption, 18-59 years**	**low consumption, 60+ years**	**high consumption, 18-59 years**	**high consumption, 60+ years**
N	**12,094**	**7,468**	**33,641**	**8,984**	**43,474**	**16,258**	**2,261**	**156**
**R^2 (%, Adjusted)**	12.83	3.36	12.49	2.73	13.51	2.37	14.37	12.53
**1.Depression (no as reference)**								
Yes	0.941 (0.682, 1.298)	1.075 (0.813, 1.420)	1.596 (1.127, 2.260)**	1.112 (0.840, 1.472)	1.238 (0.947, 1.618)	1.098 (0.900, 1.339)	6.726 (2.625, 17.233)***	0.323 (0.036, 2.865)
**2.Diatery patterns**								
Plant-based food consumption (low as reference)								
intermediate	1.101 (0.723, 1.677)	1.084 (0.747, 1.573)	1.161 (0.761, 1.772)	0.810 (0.534, 1.228)	1.198 (0.858, 1.674)	0.927 (0.693, 1.240)	0.949 (0.327, 2.758)	1.285 (0.175, 9.458)
high	1.246 (0.761, 2.039)	0.912 (0.598, 1.390)	1.049 (0.627, 1.755)	0.618 (0.380, 1.004)	1.294 (0.879, 1.904)	0.807 (0.581, 1.121)	0.119 (0.016, 0.870)*	x
Animal-based food consumption (low as reference)								

intermediate	1.318 (0.711, 2.444)	1.138 (0.763, 1.697)	1.098 (0.699, 1.726)	0.904 (0.541, 1.509)	1.122 (0.771, 1.633)	0.951 (0.680, 1.331)	0.472 (0.133, 1.668)	7.804 (0.607, 100.288)
high	0.967 (0.367, 2.552)	1.492 (0.828, 2.688)	1.019 (0.521, 1.995)	1.318 (0.671, 2.588)	0.973 (0.554, 1.710)	1.277 (0.810, 2.014)	0.129 (0.017, 0.991)*	30.968 (1.144, 838.401)*
**3. Health**								
Smoking status (never as reference)								
ever	1.202 (0.953, 1.515)	1.252 (1.048, 1.495)*	1.156 (0.920, 1.454)	1.029 (0.849, 1.247)	1.161 (0.983, 1.372)	1.108 (0.971, 1.263)	1.746 (0.746, 4.087)	1.773 (0.434, 7.251)
Alcohol consuming last month (no as reference)								
Yes	0.606 (0.471, 0.780)***	0.629 (0.499, 0.793)***	0.841 (0.666, 1.063)	0.825 (0.645, 1.056)	0.746 (0.625, 0.890)**	0.738 (0.620, 0.879)**	0.583 (0.221, 1.538)	0.388 (0.105, 1.426)
Obese (no as reference)								
Yes	2.004 (1.599, 2.511)***	1.852 (1.517, 2.261)***	2.344 (1.880, 2.923)***	1.617 (1.315, 1.989)***	2.230 (1.891, 2.629)***	1.698 (1.469, 1.962)***	1.697 (0.787, 3.661)	3.393 (0.775, 14.854)
Sufficient physical activity (Yes as reference)								
No	1.325 (1.054, 1.665)*	1.178 (0.963, 1.442)	0.887 (0.701, 1.122)	1.542 (1.217, 1.953)***	0.990 (0.834, 1.176)	1.302 (1.114, 1.520)**	0.925 (0.375, 2.279)	0.867 (0.237, 3.180)

**4.Demographic and socioeconomic status**								
Sex (male as reference)								
Female	0.806 (0.645, 1.006)	0.800 (0.666, 0.961)*	1.004 (0.800, 1.261)	0.925 (0.743, 1.153)	0.954 (0.808, 1.126)	0.879 (0.762, 1.015)	0.966 (0.420, 2.221)	1.586 (0.355, 7.084)
Region (north as reference)								
Northeast	1.048 (0.778, 1.413)	1.301 (1.013, 1.672)*	0.934 (0.684, 1.275)	1.390 (1.025, 1.884)*	0.987 (0.796, 1.224)	1.317 (1.087, 1.596)**	0.665 (0.144, 3.066)	x
Southeast	1.171 (0.834, 1.644)	1.632 (1.249, 2.131)***	1.193 (0.860, 1.655)	1.726 (1.290, 2.310)***	1.089 (0.858, 1.381)	1.464 (1.203, 1.780)***	0.631 (0.190, 2.097)	1.148 (0.188, 7.006)
South	1.195 (0.815, 1.751)	1.062 (0.784, 1.439)	1.040 (0.731, 1.481)	1.695 (1.244, 2.309)**	0.985 (0.760, 1.275)	1.223 (0.989, 1.513)	0.367 (0.085, 1.592)	0.394 (0.109, 1.427)
Central	1.267 (0.872, 1.842)	1.938 (1.372, 2.737)***	0.829 (0.583, 1.181)	1.430 (0.996, 2.051)	0.915 (0.709, 1.179)	1.526 (1.192, 1.952)**	0.382 (0.086, 1.695)	x
Highest education obtained (Elementary school- as reference)								
High school	0.699 (0.541, 0.904)**	0.743 (0.592, 0.931)*	0.750 (0.580, 0.968)*	0.798 (0.626, 1.016)	0.702 (0.581, 0.847)***	0.775 (0.657, 0.915)**	2.701 (1.122, 6.502)*	x

University+	0.505 (0.358, 0.713)***	0.635 (0.478, 0.844)**	0.482 (0.352, 0.660)***	0.695 (0.526, 0.920)*	0.466 (0.367, 0.591)***	0.674 (0.551, 0.824)***	3.032 (1.077, 8.533)*	1.825 (0.373, 8.929)
Residence (urban as reference)								
Rural	0.556 (0.426, 0.725)***	0.866 (0.693, 1.083)	0.856 (0.652, 1.124)	0.582 (0.456, 0.743)***	0.725 (0.596, 0.881)**	0.731 (0.621, 0.861)***	1.156 (0.382, 3.498)	x
Marital status (married as reference)								
Other	1.065 (0.866, 1.310)	1.058 (0.881, 1.270)	0.959 (0.765, 1.201)	0.896 (0.726, 1.107)	0.988 (0.840, 1.162)	0.969 (0.842, 1.114)	0.700 (0.320, 1.531)	x
Age	1.092 (1.074, 1.110)***	1.006 (0.995, 1.017)	1.081 (1.068, 1.095)***	1.005 (0.994, 1.016)	1.091 (1.080, 1.103)***	1.006 (0.998, 1.014)	1.057 (1.023, 1.093)**	0.350 (0.085, 1.450)

Supplementary Table S3. Results of further logistic regression.

Abbreviation: CI: confidence interval; UPF1: ultra-processed food consumption classification method 1; UPF2: ultra-processed food consumption classification method 2.

Notes

* ***P*** <0.05; ** ***P*** <0.01; ******P*** <0.001; x: observation with this value was omitted because of collinearity.
